# Gut microbiota dysbiosis affects the local renin-angiotensin system to induce osteoporosis

**DOI:** 10.3389/fendo.2025.1698719

**Published:** 2025-12-12

**Authors:** Kai Zhong, Yuanhui Wang, Pengkun Han, Zhanwen Huang, Zonghui Xie, Peigeng Yu, Hanwen Liu, Hao Hu, Meiyun Tan, Xing Guo

**Affiliations:** 1 Department of Orthopedics, Affiliated Hospital of Southwest Medical University, Luzhou, Sichuan, China; 2 Department of Nuclear Medicine, The Affiliated Hospital of Southwest Medical University, Luzhou, Sichuan, China; 3 Department of Burn and Plastic Surgery, Affiliated Hospital of Southwest Medical University, Luzhou, Sichuan, China; 4 Center of Ambulatory Surgery, Affiliated Hospital of Southwest Medical University, Luzhou, Sichuan, China

**Keywords:** gut microbiota, osteoporosis, renin-angiotensin system, RANKL, AT2R

## Abstract

**Introduction:**

The gut microbiota has been found to play a key role in bone metabolism, and renin-angiotensin components are locally expressed in bones and affect bone metabolism, but the link between the two is still unclear. This study aims to investigate whether gut microbiota dysbiosis causes osteoporosis through local renin-angiotensin system (RAS).

**Methods:**

Osteoporosis was induced in mice by long-term antibiotic feeding, which disrupted their gut microbiota. Subsequently, the role of RAS in this process was investigated by modulating angiotensin II receptor activity.

**Results:**

Long-term antibiotic treatment leads to significant alterations in gut microbiota and leads to osteoporosis, and that localized RAS in bone tissue promotes osteoclast proliferation and activity through up-regulation of ERK1/2 to promote RANKL release and inflammatory infiltration, leading to osteoporosis, and that the angiotensin II Type 2 Receptor (AT2R) may be an important target for this process.

**Conclusions:**

Dysbiosis of the gut microbiota leads to osteoporosis by enhancing the activity of the local renin-angiotensin system in bones. Upregulation of ERK1/2 and RANKL, along with inflammatory responses, collectively drive bone loss. AT2R may be a potential therapeutic target. These findings highlight the role of the gut-bone axis and suggest that targeted therapeutic approaches should be further investigated.

## Introduction

The gut microbiota is a dynamic and complex microbial community that has been shown to closely correlate with the host, influencing the inflammatory response, intestinal physiology, immune function, and metabolic activity (1–4). Since Sjögren (5) et al. first identified the influence of the gut microbiota on bone density, numerous studies have confirmed the gut-bone axis connection and expanded understanding of its potential mechanisms (6).

Differences in bone density and trabecular microarchitecture between germ-free and wild-type mice highlight the influence of gut microbiota on bone metabolism (5). Pathogenic bacteria-induced colitis increases bone loss in mice, and probiotic treatment has been shown to attenuate bone loss associated with ovarian degeneration in female mice and type 1 diabetes in male mice (7–10). These findings underscore the role of the gut microbiota in bone metabolism and its critical importance for bone growth and development in mice. Furthermore, dysregulation of the gut microbiota may disrupt normal bone development. Despite these findings, the exact mechanisms by which the gut microbiota affects bone metabolism are still poorly understood. It is well known that dysbiosis of the gut microbiota alters systemic inflammatory and hormonal pathways. Still, its specific effects on bone tissue and the molecular mediators involved must be further elucidated. The renin-angiotensin system is a potential candidate for linking the gut microbiota to bone health (11).

The renin-angiotensin system is a key endocrine pathway that regulates blood pressure, fluid homeostasis, and cardiovascular function through its major effector molecule, angiotensin II (Ang II) (12). In addition to their systemic roles, recent studies have found that RAS components, including angiotensin II and its receptors (AT1R and AT2R), are expressed in bone tissue, where they regulate osteoblast and osteoclast activity (13). Abnormal localized RAS activation in bone tissue has been shown to lead to bone loss and impaired bone remodeling and is associated with multiple factors leading to osteoporosis (13–15). However, the link between gut microbiota dysbiosis and local RAS activation in bone tissue has not been thoroughly investigated.

We hypothesize that gut microbiota dysbiosis induced by long-term antibiotic treatment activates local RAS in bone tissue, leading to osteoporosis. This study aimed to investigate the effects of dysbiosis on bone metabolism, the role of RAS in this process, and the potential molecular mechanisms linking the gut microbiota to bone health.

## Method

### Animals

Sixty 4-week-old male Kunming mice were obtained from the Laboratory Animal Center of Southwest Medical University. They were housed in an SPF environment with a 12-hour light cycle and provided free access to food and water. The mice were randomly divided into a control group (n=12) and an experimental group (n=48). Starting at 4 weeks of age, the experimental group’s drinking water was supplemented with broad-spectrum antibiotics (neomycin 0.5 g/L, ampicillin 1.0 g/L). Both the experimental and control groups had their drinking water refreshed every two days, with the experimental group receiving water containing antibiotics at each. After 12 weeks of treatment, bone mineral density at the lumbar spine (L3-4) was measured using dual-energy X-ray absorptiometry to elucidate the effects of antibiotic treatment on bone metabolism and establish a post-dysbiosis osteoporosis model.

Following model confirmation, mice were randomly assigned to the losartan group (n=12), PD123319 group (n=12), and CGP42112A group (n=12). They received oral administration via gastric tube of 10 mg/kg losartan potassium, 15 μg/kg/day PD123319, and intraperitoneal injection of CGP42112A, respectively. All mice concurrently received 0.5 ml saline via gastric tube or intraperitoneal injection. All anesthetic procedures employed 2.5% isoflurane inhalation anesthesia, and euthanasia was performed via cervical dislocation under anesthesia. Treatment lasted for four weeks. All animal experiments were approved by the Animal Research and Care Committee of Southwest Medical University.

### Gut microbiota 16S rRNA sequencing

After 12 weeks of feeding, feces were collected from all mice using sterile tubes with at least two feces per mouse (>0.1g/serving) and stored immediately at -80 °C. Fecal samples from 8 mice in the experimental and control groups were randomly selected for 16S rRNA sequencing using MiSeq technology (16). Briefly, DNA was extracted and the 16S ‘ V4-V5’ region was amplified using specific primers (515F 5’-GTG CCA GCM GCCGCG GTAA-3’; 926R 5’-CCG TCA ATT CMT TTG AGT TT-3’). The amplified products were purified and quantified to create a 16S rRNA library and sequenced (Illumina). Sequences were then quality trimmed to identify and remove chimeric sequences (Trimmomatic and mothur), and sequences were classified using USEARCH software to remove those classified as eukaryotic, archaeal, chloroplast, mitochondrial, or unknown. Finally, the sequence data were filtered to remove any sequences that appeared only once in the dataset, and the clean tags processed above were clustered OTUs, and the sequences were clustered to operational taxonomic units (OTUs) with a 97% similarity using USEARCH. The community richness and diversity were analyzed by Mothur to explore the alpha diversity of mouse gut microbiota, and the species richness estimators were assessed using the ACE estimator and Chao estimator, while species diversity were evaluated using the Shannon estimator and Simpson estimator. To visually investigate the similarity or difference of the data, the eigenvalues and eigenvectors between samples were determined by calculating ecological distances (beta diversity) to perform a principal coordinate analysis (PCoA) based on OTUs and evolutionary relationships, and to create a matrix heat map.

Analysis of species differences across levels was conducted by normalizing relative species abundance based on OTUs of each sample to 30,000 sequences, followed by Wilcoxon signed-rank test to assess differences between two groups.

### Bone mineral density measurement

BMD of lumbar vertebrae (L3-4) in mice was measured by dual-energy X-ray scanning (GE Healthcare, Piscataway, NJ, USA). Mice were anesthetized and immobilized on a test bench and scanned at a speed of 1 mm/sec with a resolution of 0.5 x 0.5 mm. The average bone density of the L3 and L4 vertebrae is considered to be an indicator of the overall bone density of the lumbar spine.

### HE staining

After 12 weeks of antibiotic treatment, mice were euthanized to collect femoral specimens, and the specimens were washed with saline after removal of soft tissues. Subsequently, decalcification was performed using 10% ethylenediaminetetraacetic acid (EDTA) solution at room temperature, and the solution was changed every 3 days. After decalcification, the specimens were embedded in paraffin, sectioned at a thickness of 3 μm, and stained with hematoxylin-eosin (H&E). Subsequently, histomorphology analysis of the distal femur was performed.

### RT-qPCR

Total RNA was isolated from cells and bone tissues using Trizol reagent and reverse transcribed using the TOYOBO ReverTra Ace qPCR RT Kit (TOYOBO, Osaka, Japan) according to the manufacturer’s instructions. RT-qPCR was then performed on a QuantStudio 3 Sequence Detection System (Applied Biosystems, USA). The sequences of the oligonucleotide primers are summarized in **Table 1**. GAPDH was used as a reference gene. The relative expression levels of the target genes were determined using the comparative ΔΔCt method.

**Table 1:** Nucleotide sequences for real-time quantitative RT-PCR primers.

Target genes	Sequences of primers (5′-3′)
RANKL	Forward: GTGAAGACACACTACCTGACTCC
	Reverse: GCCACATCCAACCATGAGCCTT
OPG	Forward: CGGAAACAGAGAAGCCACGCAA
	Reverse: CTGTCCACCAAAACACTCAGCC
AT1R	Forward: GCCATTGTCCACCCGATGAAGT
	Reverse: ACACATTTCGGTGGATGACGGC
AT2R	Forward: CGTGACCAAGTCCTGAAGATGG
	Reverse: GGAAGTGCCAGGTCAATGACTG
IL-17	Forward: CAGACTACCTCAACCGTTCCAC
	Reverse: TCCAGCTTTCCCTCCGCATTGA
TGF-β	Forward: CCACCTGCAAGACCATCGAC
	Reverse: CTGGCGAGCCTTAGTTTGGAC
GAPDH	Forward: GTATGAAGTGCCCCTCCTTG
	Reverse: CCCCAGGCTGTACAAGACAT

### Cell extraction and culture

Bone marrow cells were derived from the tibia and femur of 4-week-old mice and cultured overnight in DMEM medium containing 10% fetal bovine serum. Subsequently, non-adherent cells were collected and induced to differentiate into osteoclasts in α-MEM medium with 100ng/ml RANKL, 50ng/ml M-CSF, and 10% fetal bovine serum.

Bone marrow cells were induced to differentiate into osteoblasts in DMEM medium containing 10% fetal bovine serum, 0.2% ascorbic acid, 1% sodium β-glycerophosphate, and 0.01% dexamethasone. And the co-culture system was performed using a 0.4 μm transwell insert. Briefly, cells intended for osteoclast differentiation were cultured in the lower well plate and supplemented with osteoclast differentiation induction medium. Cells intended for osteoblast induction were cultured in the upper transwell insert and supplemented with osteoblast differentiation induction medium. After pre-induction for 5 days, the transwell insert was inserted into the well plate to initiate co-culture. For osteoblast evaluation, the cells in the upper and lower layers are swapped, and no pre-induction is performed. Angiotensin II is added to the culture media in both the upper and lower layers. Ang II was added to both the upper and lower layers.

Ang II was added to the culture medium after the optimal concentration was clarified by MTT assay.

After 14 days of osteogenic differentiation or 7 days of osteoblastic differentiation, the cells were harvested for further biochemical tests.

### MTT assay

Osteoblasts and osteoclasts were cultured in basal medium with varying concentrations of Ang II (10^-4^- 10–^8^ M) for 2 days. After replacing the medium, an equal amount of MTT solution (0.5 mg/mL) was added and incubated at 37 °C for 4h to form formazan crystals. The OD value was recorded at 490 nm after adding the dissolution solution, and the result was calculated.

### Alkaline phosphatase staining

Osteoblasts were fixed in 4% paraformaldehyde at 4 °C, washed in PBS, and stained using an alkaline phosphatase staining kit for 30 minutes at room temperature (Beyotime C3250S), and photographed for microscopic observation.

### Anti-tartaric acid phosphatase stain

Osteoblasts were fixed in 4% paraformaldehyde at 4 °C, washed in PBS, stained using the TRAP staining kit for 1 hour at 37 °C (Sigma 387A), and photographed for microscopic observation.

### Bone resorption experiment

Bovine bone slices of 200 μm thickness were washed using complete culture and placed in 96-well plates. Osteoclast precursors were inoculated onto the surface of bovine bone slices and induced osteoclast differentiation, and co-cultures were performed using HTS Transwell 96 well permeable supports (Corning 3382).

### ELISA

Mice were blood sampled from the heart at 16 and 20 weeks of age under anesthesia for 1 ml, and serum was collected by centrifugation, followed by the Mouse Angiotensin II (Ang II) and Nuclear Factor κ B Receptor Activating Factor Ligand (RANKL) ELISA Kit (Elabscience Biotechnology, Inc.) at room temperature according to the manufacturer’s instructions. Briefly, during testing, add 100 µL each of the standard (0–10 ng/mL serial dilutions), blank, and sample to the coated plate wells. Incubate at 37 °C for 90 min. After discarding the wells, add 100 µL of biotinylated detection antibody and incubate at 37 °C for 1 h. Wash the plate three times, then add 100 µL of HRP-conjugated streptavidin and incubate at 37 °C for 30 min. After washing the plate five more times, add 90 µL of TMB substrate and incubate in the dark for approximately 15 minutes. Add 50 µL of stop solution to terminate the reaction, then read the OD value at 450 nm (duplication recommended). Calculate the sample concentration using a four-parameter logistic (4-PL) fit of the OD value corrected for the blank against the standard concentration.

### Micro-CT

Mice were executed at 20 weeks of age, and femoral samples were collected. High-resolution scans were performed using a Siemens Inveon Micro-PET/CT system with a resolution of 20 μm. Each scan included the specimen and a calibration model to standardize gray-scale values and maintain consistency between analyses. Bone marrow was separated from bone using a fixed threshold of 980, and areas of interest were identified using hand-drawn contour lines in the region of trabeculae 1-1.5 mm from the proximal femoral growth plate. Data were analyzed and documented using the Inveon Research Workplace software (Siemens), which analyzes bone volume fraction (BV/TV), trabecular thickness (Tb.Th), trabecular number (Tb.N), and trabecular volume (Tb.N). number (Tb.N) and trabecular spacing (Tb.Sp). All skeletal analyses were performed without regard to specific experimental conditions.

### Western blot

Proteins in bone tissue were extracted using RIPA buffer (Solarbio) containing protease inhibitor (Roche) and phosphatase inhibitor (Roche). Twenty micrograms of total protein were loaded onto a 10% SDS-PAGE gel and transferred to a PVDF membrane. NF-ĸB p65, ERK1/2, and GAPDH (Abcam) were detected using specific primary antibodies. After 2 h of incubation in secondary antibody (Affinity), the bands were color developed using the ECL kit.

### Statistical analysis

Statistical analysis was performed using GraphPad Prism. For data that conformed to a normal distribution, a t-test was used for comparisons between two groups, and an analysis of variance (ANOVA) was used for comparisons between three groups.

For data that were not normally distributed, the Wilcoxon test was used for comparisons between two groups. P < 0.05 is considered to be statistically different.

## Results

### Antibiotic treatment had no significant effect on body weight but increased body fat percentage in mice

To clarify the effects of gut microbiota dysbiosis on bone metabolism and the role of the RAS in this process, we treated mice with continuous antibiotics for 12 weeks to disrupt the gut microbiota.

**Figure 1:**
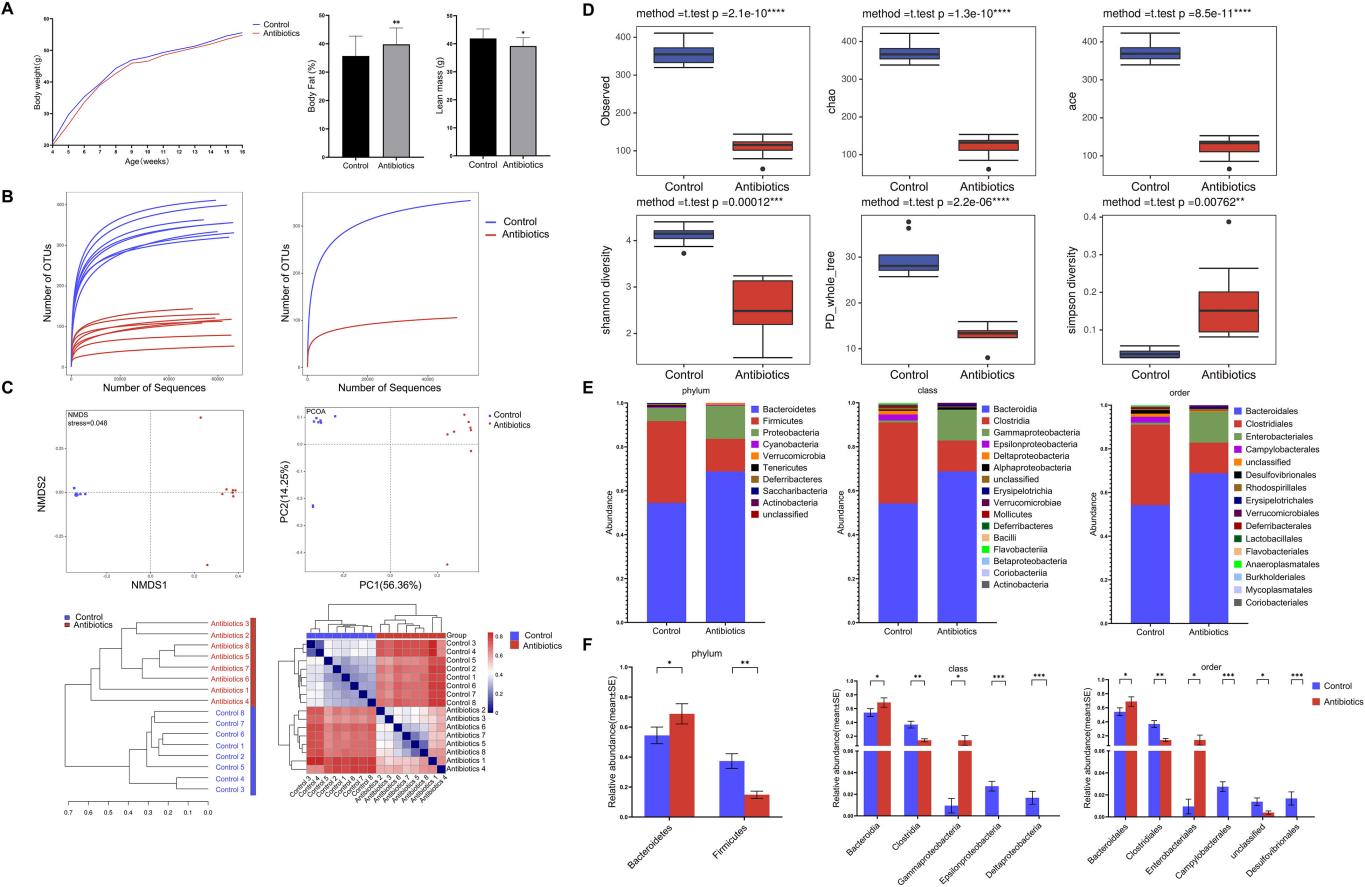
Antibiotic treatment leads to increased body fat percentage and gut microbiota dysbiosis in mice. **(A)** Body weight trends in two groups of mice from 4 to 16 weeks of age, and comparison of body fat percentage or lean body mass between groups at 16 weeks; **(B)** 16S rRNA sequencing of mouse fecal samples at 16 weeks of age; multiple samples and averaged dilution curves confirm adequate sequencing depth. **(C)** Evolutionary-based β-diversity analysis comparing species diversity differences between groups, including unweighted Unifrac NMDS analysis (top left), unweighted Unifrac PCoA analysis (top right), unweighted Unifrac multi-sample similarity tree analysis (bottom left), and unweighted Unifrac differential matrix heatmap (bottom right);**(D)** α diversity analysis evaluated microbial community abundance and diversity between groups, including Observed index, Chao index, HAO index, ACE index, Shannon index, Simpson index, and PD_whole_tree index; **(E)** Bar charts showing community structure composition at different levels between groups; **(F)** Comparison of relative abundance of key taxonomic units at each level. *p<0.05, **p<0.01, ***p<0.001.

Since bone mass in mice is strongly correlated with body weight, we monitored the effect of antibiotic treatment on body weight in mice. After 12 weeks of antibiotic treatment, the total weight of the mice remained within the expected growth range, with no significant difference between the two groups. Interestingly, antibiotic treatment resulted in a significant increase in adiposity and a significant decrease in lean body mass in mice (**Figure 1A**).

### The antibiotic treatment causes dysbiosis of the gut microbiota in mice

To investigate the effects of antibiotic treatment on the gut microbiota of mice, we performed 16S rRNA sequencing of mouse fecal samples to assess changes in the gut microbiota. First, we constructed Rarefaction curves and found that the Rarefaction curves leveled off at the end of individual samples and the overall level, and the sequencing depth was reasonable for subsequent studies (**Figure 1B**). Subsequently, β-diversity analysis was performed on the samples, including heat maps, PCoA, and similarity clustering diagrams, which showed significant clustering among samples in the same group, significant separation between samples in different groups, and good reproducibility of the samples (**Figure 1C**).

Subsequently, we performed α diversity analysis and found that the observation index, Chao index, ACE index, Shannon index, and PD whole tree index of the mouse gut microbiota significantly decreased after antibiotic treatment, while the Simpson index significantly increased, this indicates that antibiotic treatment led to reduced species richness (Chao, ACE) and species diversity (Shannon, Simpson) in the mouse gut microbiota (**Figure 1D**).

To identify which species of gut microbiota were affected by antibiotic treatment, we analyzed the composition of the gut microbiota at the Phylum, Class, and Order levels and identified the most significantly altered species by nonparametric tests. Antibiotic treatment significantly increased the relative abundance of *Bacteroidetes* and decreased the relative abundance of *Firmicutes* at the Phylum level; significantly increased the relative abundance of *Bacteroidia* and decreased the relative abundance of *Clostridia* at the Class level; and significantly increased the relative abundance of *Bacteroidales* and decreased the relative abundance of *Clostridiales* at the Order level (**Figure 1E**). To identify species exhibiting the most significant differences across levels, relative abundances were normalized and a Wilcoxon signed-rank test was performed. This revealed that the previously mentioned species showed the most pronounced changes at their corresponding levels (**Figure 1F**).

These data suggest that antibiotic therapy can disrupt the structure of the gut microbiota and lead to gut microbiota dysbiosis.

### Gut microbiota dysbiosis may promote osteoporosis by affecting the RAS

**Figure 2:**
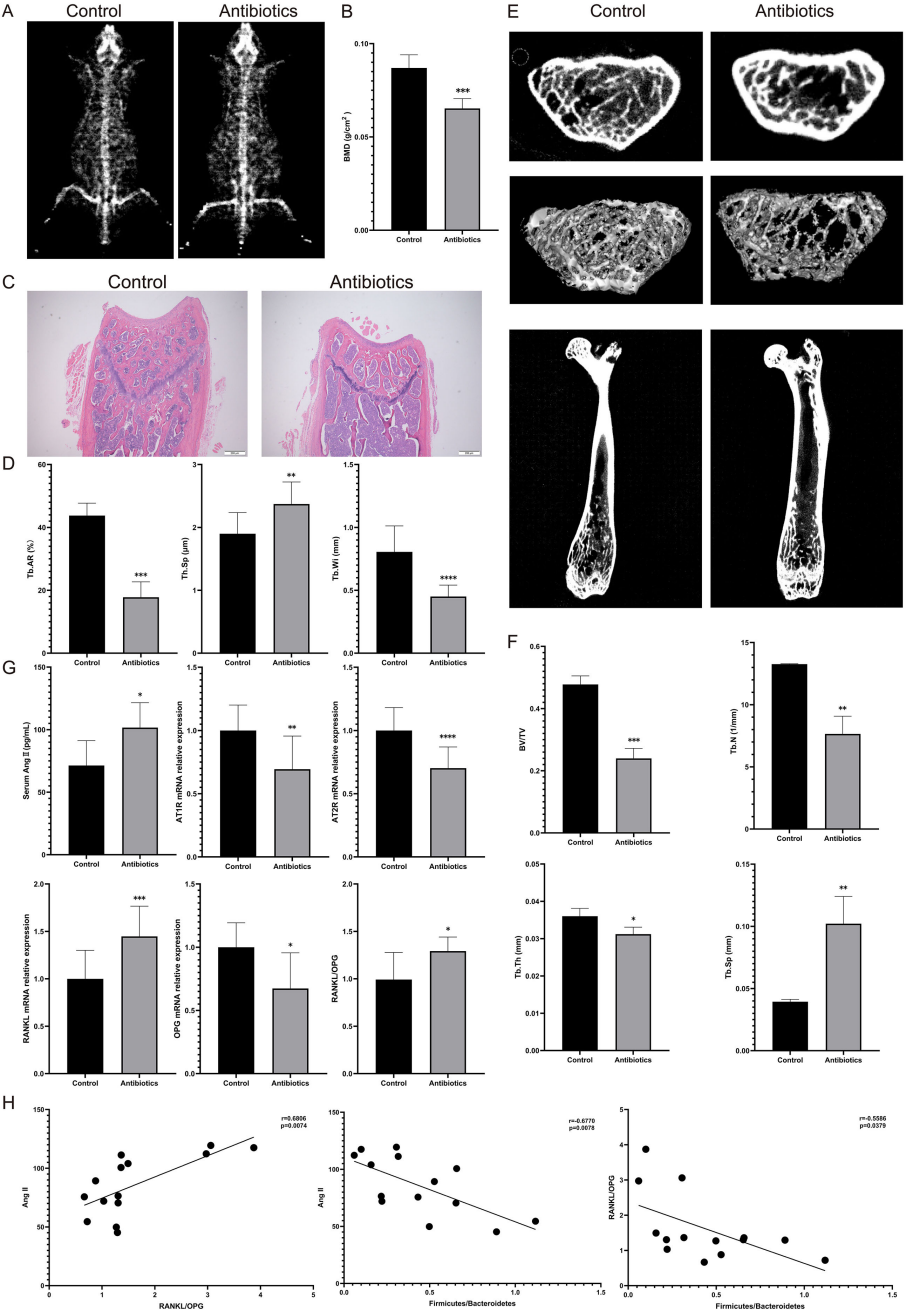
Antibiotic treatment causes osteoporosis in mice and activates local RAS in bone tissue. Antibiotic treatment induces osteoporosis in mice and activates the local renin-angiotensin system within bone tissue. **(A)** Representative dual-energy X-ray absorptiometry (DXA) images at 16 weeks of age for control and antibiotic groups; **(B)** Comparison of bone mineral density (BMD) between groups based on DXA; **(C)** Representative hematoxylin and eosin (HE) stained images of distal femurs; **(D)** Comparison of trabecular bone area (Tb. Ar), trabecular bone width (Tb. Wi), and trabecular bone spacing (Tb. Sp) between groups; **(E)** Representative micro-CT images at 16 weeks of age; **(F)** Quantitative analysis of bone volume/total volume ratio (BV/TV), trabecular number (Tb. N), trabecular thickness (Tb. Th), and trabecular spacing (Tb. Sp); **(G)** At 16 weeks of age, serum angiotensin II (Ang II) levels and mRNA expression levels of AT1R, AT2R, RANKL, and OPG in bone tissue, as well as the RANKL/OPG mRNA expression ratio; **(H)** Correlation analysis between serum Ang II levels and RANKL/OPG (left), Firmicutes/Bacteroidetes ratio and serum Ang II levels (middle), and RANKL/OPG ratio (right). *p<0.05, **p<0.01, ***p<0.001, ****p<0.0001.

To clarify the effect of gut microbiota dysbiosis on bone quality in mice, we first assessed the mean BMD of mice at L3-L4 by DXA, and found that BMD was significantly lower in mice after gut microbiota dysbiosis (**Figures 2A, B**). Then, we performed histomorphology analysis of distal femoral trabeculae by HE staining, and found that trabeculae were significantly fewer in mice after gut microbiota dysbiosis, and quantitative analysis showed that Tb. Ar and Tb. Wi was significantly decreased and Tb. Sp was significantly increased (**Figures 2C, D**). Subsequently, we assessed distal femoral trabecular microarchitecture by micro-CT. Consistent with the histomorphometry, gut microbiota dysbiosis reduced trabecular number and markedly deteriorated architecture showed a decrease in BV/TV, Tb.Th, and Tb.N, while Tb.Sp increased (**Figures 2E, F**).

These data suggest that gut microbiota dysbiosis induced by antibiotic therapy can lead to significant bone destruction and cause osteoporosis.

To investigate the link between gut microbiota dysbiosis and RAS, we measured the main effector protein Ang II. The levels of Ang II were found to be significantly increased in the serum of mice with gut microbiota dysbiosis. To further clarify the changes of RAS in bone tissues, we measured the expression levels of AT1R and AT2R, receptors of Ang II, which were found to be significantly increased in bone tissues after gut microbiota dysbiosis. In addition, RANKL expression was significantly increased, OPG expression was significantly decreased, and the RANKL/OPG ratio was significantly increased in bone tissues after gut microbiota dysbiosis (**Figure 2G**). Correlation analysis revealed that the Firmicutes/Bacteroidetes ratio showed a significant negative correlation with Ang II levels (r = 0.6770, p = 0.0078) and the RANKL/OPG ratio (r = -0.5586, p = 0.0379). Simultaneously, Ang II levels were positively correlated with the RANKL/OPG ratio (r = 0.6806, p=0.0074) (**Figure 2H**).

These results suggest that the dysbiosis of the gut microbiota caused by antibiotic treatment may contribute to the development of osteoporosis by affecting the RAS.

### Ang II promotes osteoclast proliferation and differentiation through indirect effects

**Figure 3:**
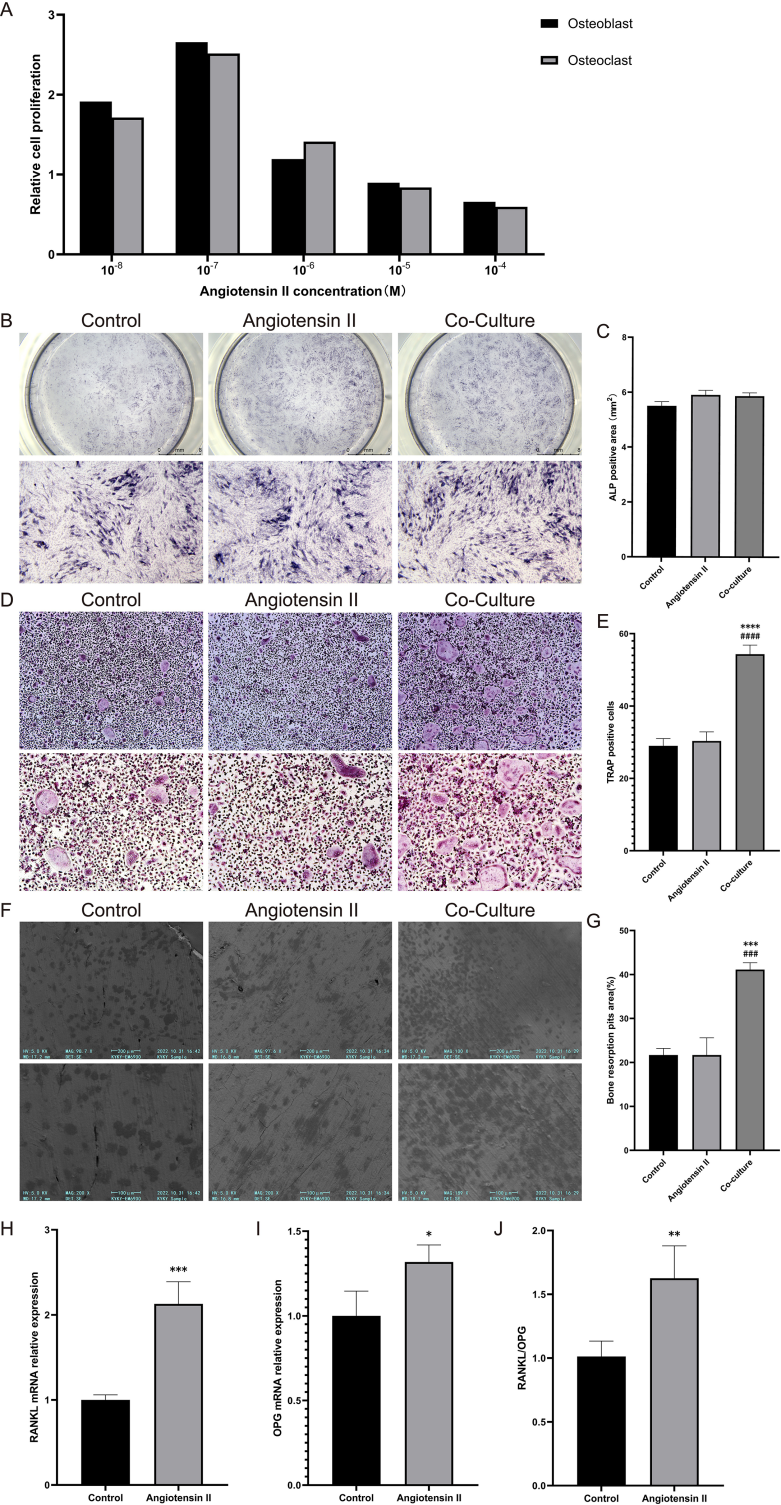
Angiotensin II indirectly promotes osteoclast formation and bone resorption by acting on osteoblasts to enhance RANKL release. **(A)** MTT assay measuring angiotensin II’s effect on cell proliferation. **(B, C)** Representative microscopic images of osteoblast ALP staining at 14 days post-differentiation induction and corresponding statistical analysis of positive areas; **(D, E)** Osteoclast Trap staining, representative microscopic images, and corresponding positive cell count statistical analysis; **(F, G)** Representative SEM images of osteoclast bone resorption experiments and corresponding statistical analysis of resorption pits; **(H–J)** qPCR measurement of RANKL and OPG mRNA expression in osteoblasts and corresponding RANKL/OPG mRNA expression ratios. Staining in the co-culture system is performed only on the lower-layer well plate, not on the upper-layer Transwell insert. Compared with the control group *p<0.05, **p<0.01, ***p<0.001, ****p<0.0001. Compared with the antibiotic group ###p<0.001, ####p<0.0001.

In order to investigate the mechanism of bone loss due to local RAS activation, we added Ang II to cell culture *in vitro* to mimic the RAS-activated environment. We firstly measured cell proliferation by MTT assay to clarify the effect of Ang II on cell activity and to determine its optimal concentration. The effect of Ang II on the proliferation of osteoblasts and osteoclasts in the concentration range of 10^-8^M-10^-4^M showed a dose-dependent effect, with the concentration of Ang II less than 10^-6^M promoting cell proliferation and that of Ang II more than 10-6M inhibiting cell proliferation. At a concentration of 10-6M, Ang II had no significant effect on the proliferation of osteoblasts and osteoclasts (**Figure 3A**). Therefore, 10^-6^M was used in the subsequent *in vitro* studies.

To investigate the effect of Ang II on bone metabolism, we treated osteoblasts, osteoclasts, and Transwell co-cultures with Ang II. The osteoblast differentiation First, we assessed the effect of Ang II on osteoblast differentiation in osteoblast cultures via ALP staining. Compared to the control group, Ang II showed no significant impact on osteoblast differentiation in either the monoculture or co-culture systems (**Figures 3B, C**). Subsequently, we assessed the effect of Ang II on osteoclast differentiation via Trap staining and found a significant increase in Trap-positive multinucleated cells in the co-culture system, whereas no such change was observed in the monoculture system (**Figures 3D, E**). These findings suggest that Ang II may promote osteoclast differentiation through indirect mechanisms. We further assessed the impact of Ang II on osteoclast resorptive capacity through bone resorption assays. Similar to the Trap staining, Ang II significantly increased both the number and area of resorption pits in the co-culture system but not in monoculture (**Figures 3F, G**). This finding further confirmed the role of Ang II in promoting osteoclast differentiation and bone resorption.

Accordingly, we hypothesize that Ang II may promote osteoclast differentiation by affecting osteoblasts. Therefore, an evaluation was conducted of the mRNA expression of RANKL and OPG in osteoblasts, and found that both were significantly increased after Ang II treatment, with a markedly elevated RANKL/OPG ratio. This suggests that Ang II influences osteoblasts to increase RANKL expression and secretion (**Figures 3H–J**).

These data indicate that Ang II indirectly promotes osteoclast differentiation and bone resorption by enhancing osteoblast expression and secretion of RANKL.

### AT2R plays a key role in osteoporosis following gut microbiota dysbiosis

**Figure 4:**
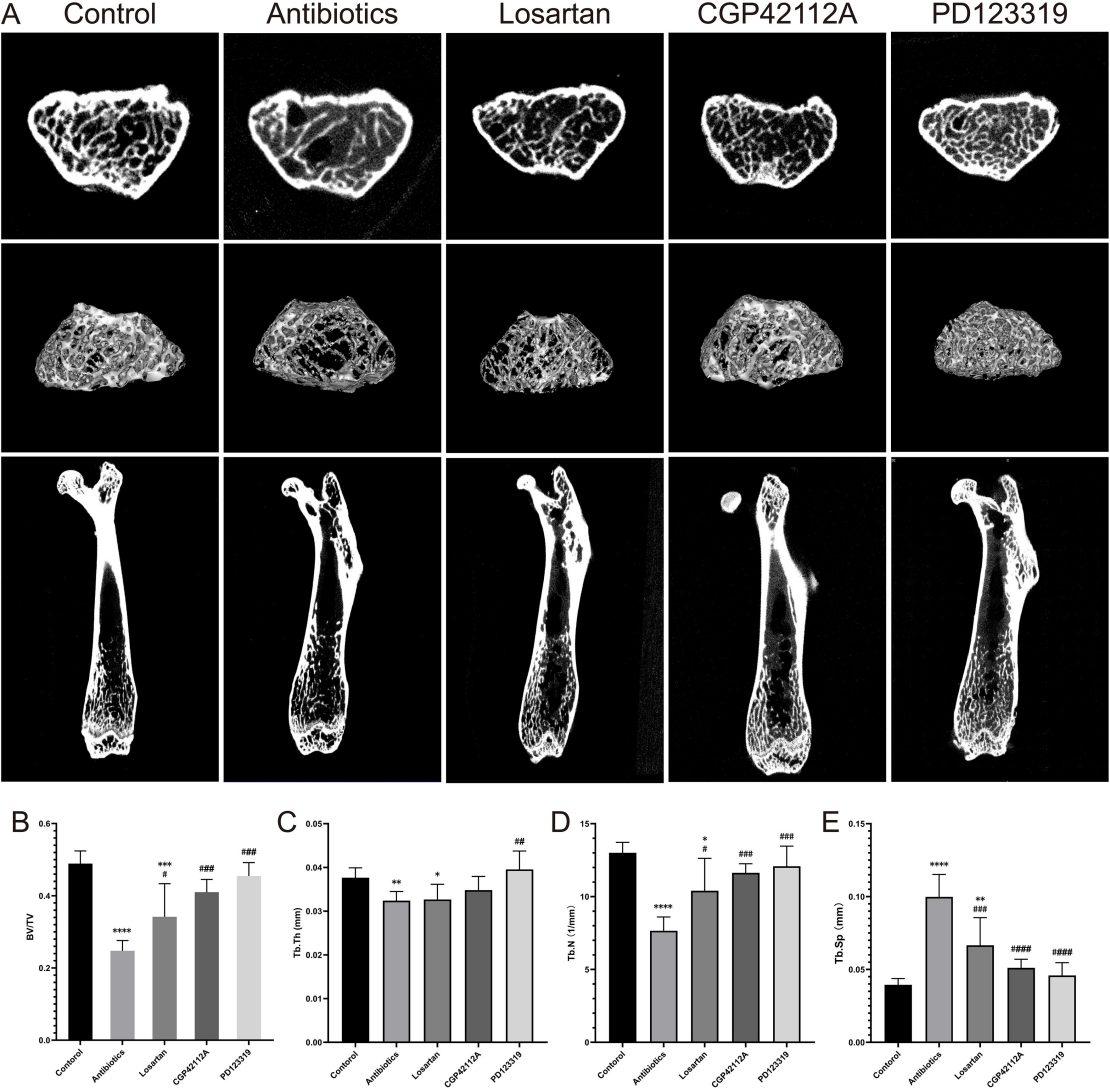
Modulation of angiotensin receptors significantly improves antibiotic-induced bone loss and structural deterioration. **(A)** Representative Micro-CT images at 20 weeks of age, from left to right: Control, antibiotic group, losartan group, CGP42112A group, and PD123319 group. **(B–E)** Micro-CT-based analysis of trabecular microstructural parameters, including bone volume/total volume ratio (BV/TV), trabecular number (Tb. N), trabecular thickness (Tb. Th), and trabecular spacing (Tb. Sp). Compared with the control group *p<0.05, **p<0.01, ***p<0.001, ****p<0.0001. Compared with the antibiotic group ##p<0.01, ###p<0.001, ####p<0.0001.

To clarify the target of RAS in osteoporosis after gut microbiota dysbiosis, we treated losartan (AT1R inhibitor), PD123319 (AT2R inhibitor), and CGP42112A (AT2R agonist) for 4 weeks after the diagnosis of osteoporosis (**Figure 4A**). It was found that losartan, PD123319, and CGP42112A all significantly increased BV/TV, Tb. Th and Tb.N, while Tb.Sp significantly decreased (**Figures 4B–E**), alleviating osteoporosis symptoms caused by gut microbiota dysbiosis. Notably, the efficacy of PD123319 was significantly better than that of losartan and CGP42112A.

These results suggest that AT2R may be an important target for localized RAS activation in bone tissue following gut microbiota dysbiosis, leading to osteoporosis.

### Dysbiosis of the gut microbiota induces osteoporosis by activating local RAS in bone tissue

**Figure 5:**
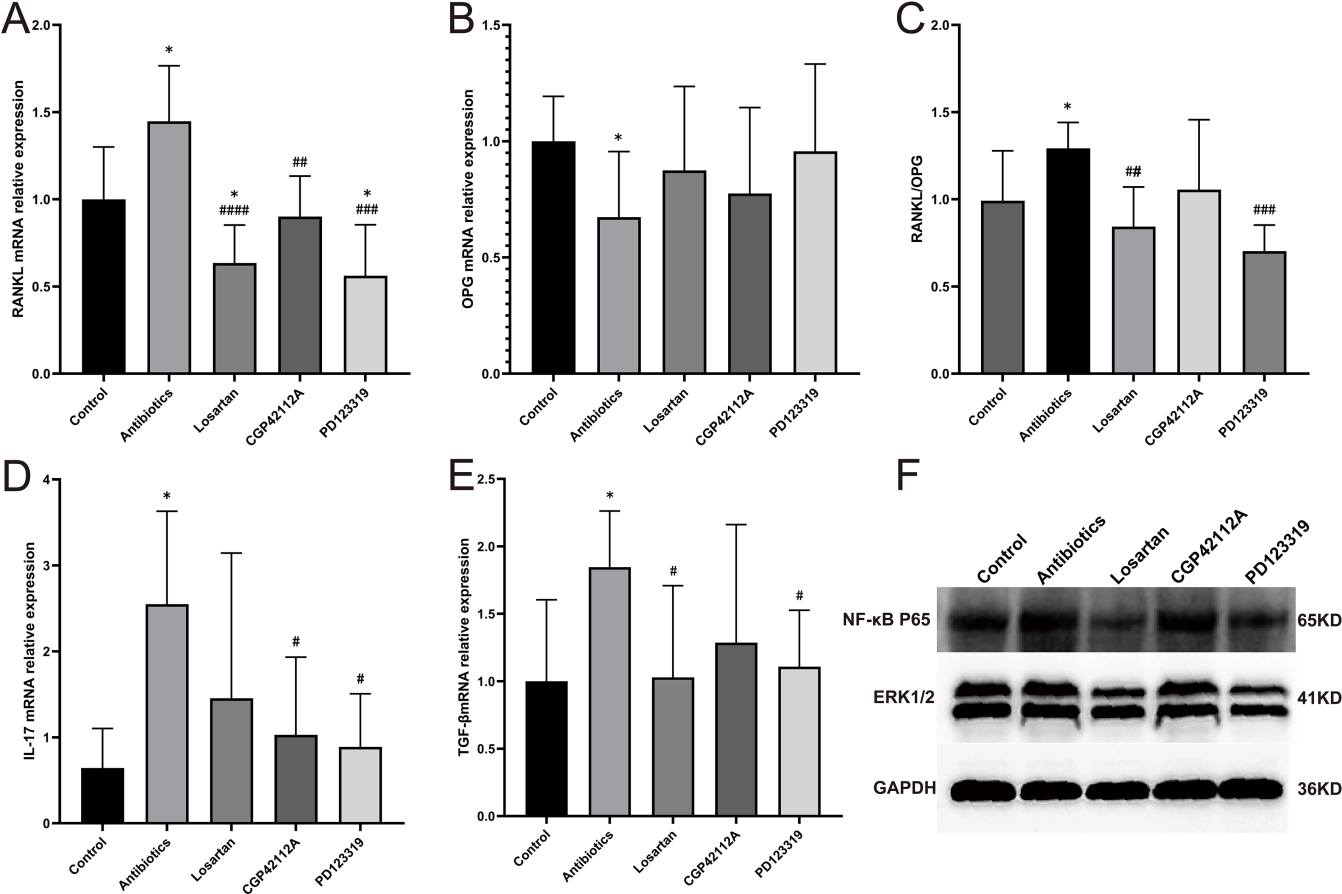
Local RAS in bone tissue promotes osteoporosis by enhancing the RANKL-RANK signaling cascade and modulating the local inflammatory microenvironment. **(A)** RANKL mRNA expression in bone tissue; **(B)** OPG mRNA expression in bone tissue; **(C)** RANKL/OPG mRNA expression ratio in bone tissue; **(D)** IL-17 mRNA expression in bone tissue; **(E)** TGF-β mRNA expression in bone tissue; **(F)** Western blot analysis of ERK1/2 and NF-κB p65 protein expression in bone tissue. All samples were collected from 20-week-old mice. Compared with the control group, *p<0.05. Compared with the antibiotic group #p<0.05 ##p<0.01, ###p<0.001, ####p<0.0001.

To clarify how the gut microbiota dysbiosis leads to the development of osteoporosis via localized RAS in bone tissues, we first determined the expression of RANKL and OPG in bone tissues and calculated their ratios. We first measured the expression of RANKL and OPG in bone tissue and the ratio of the two. The same results as in the previous results showed that the expression of RANKL was significantly increased and the expression of OPG was significantly decreased in mice with gut microbiota dysbiosis after antibiotic treatment, and the expression of RANKL/OPG was significantly increased (**Figures 5A–C**), which suggests that osteoporosis is related to the enhancement of osteoclast activity. After treatment with losartan, PD123319, and CGP42112A, RANKL expression was significantly decreased, OPG expression was slightly increased, and RANKL/OPG was significantly decreased (**Figure 5A, C**), which further suggests that RANKL-mediated osteoclast activity plays an important role in the development of osteoporosis. In addition, the inflammatory response after RAS activation is also closely related to osteoclasts, therefore, we examined the expression of IL-17 and TGF-β in bone tissues, and found that the expression of IL-17 and TGF-β was significantly increased in mice with dysbiosis of gut microbiota after antibiotic treatment, and the treatment of losartan, PD123319 and CGP42112A could rescue this phenotype (**Figures 5D, E**).

Subsequently, we determined the protein expression in bone tissues of NF-κB P65, a key target in the RANKL-RANK signaling cascade, and ERK1/2, a key molecule in RAS-activated inflammation, and the changes of both were similar to those of RANKL and IL-17 mRNA levels (**Figure 5F**).

These results suggest that dysregulation of the gut microbiota activates local RAS-regulated inflammation and RANKL-RANK signaling cascade in bone tissue to promote osteoclast activity, leading to osteoporosis.

## Discussion

Our study showed that 12 consecutive weeks of antibiotic treatment led to gut microbiota dysregulation in mice, resulting in deterioration of bone microarchitecture and the development of osteoporosis. And through *in vitro* and *in vivo* studies, we found that local RAS in bone tissue plays an important role in this process by regulating inflammation and RANKL-RANK signaling cascade to promote osteoclast activity through AT2R.

Studies have shown that the gut microbiota can have a significant impact on a multitude of host systems (1–4), and while the sterile mouse model confirms the existence of an important role for the gut microbiota in bone metabolism, the immunodeficiency of this model cannot be ignored (5). Therefore, it is more reasonable to explore the role of gut microbiota in bone metabolism by perturbing it (17, 18). Relevant studies have shown that antibiotic treatment leads to a massive depletion of commensal bacteria, resulting in long-term alterations of the gut microbiota and dysbiosis, which in turn have lasting negative effects on the host (19). While studies, including ours, have found significant alterations in bone mineral density in mice after antibiotic treatment, it is worth noting that the results were inconsistent across studies due to the differences in the dose, type, and duration of antibiotics (7, 20, 21). This may be related to the resistance of the gut microbiota to external factors, and short-term antibiotic treatment cannot change the composition of the gut microbiota in the long term (22). In addition, the age, sex, and strain of mice are not negligible factors, such as COX (21) et al. found that low-dose penicillin treatment increased bone density in male mice and decreased it in females. Therefore, in this study, we applied long-term antibiotic treatment with ampicillin and neomycin sulfate, which have very low absorption rates in the intestinal tract of mice, to achieve long-term interference with the composition of the gut microbiota and to reduce the parenteral effects of antibiotic treatment (23–25).However, the systemic effects of antibiotic treatment cannot be overlooked. These include alterations in physical activity, changes in short-chain fatty acid (SCFAs) levels associated with gut microbiota dysbiosis, and impacts on other crucial signaling pathways such as the BMP pathway.

In the present study, 16S rRNA sequencing of feces confirmed that antibiotic treatment had a significant effect on the composition of the gut microbiota in mice at multiple levels from Phylum to Order, similar to other studies (17, 18, 20, 26). But interestingly, the relative abundance of *Bacteroidetes* was significantly increased in the present study whereas other studies have found that antibiotic treatment resulted in a significant decrease in the relative abundance of *Bacteroidetes* and the decrease in the abundance of it has been suggested to be related to the development of diseases including intestinal disorders and type 2 diabetes (27, 28). We speculate that the differences in Bacteroidetes alterations may be related to variations in diet and antibiotic type and dosage. In this study, we found that there was a significant decrease in the relative abundance of *Lactobacillus* in the *Firmicutes* phylum, which was suggested to be a probiotic that is beneficial to bone health in related study (11, 29). Thus, we postulated that the reduced abundance of *Lactobacillus* was related to the occurrence of osteoporosis.

Related studies have confirmed the link between gut microbiota and RAS through a rat model of spontaneous hypertension (30). However, it is unclear how the gut microbiota affects RAS activity. In the present study, we observed that antibiotic treatment led to a reduction in the *Firmicutes/Bacteroidetes* ratio, which showed a significant negative correlation with Ang II levels, and RANKL/OPG ratio, similar to literature (30),suggesting the potential involvement of the RAS in regulating the gut-bone axis. And a decrease in the relative abundance of *Lactobacillus* and an increase in Ang II levels were observed, whereas *Lactobacillus* has been found to produce valine-proline (VPP) and the biologically active peptide isoleucine-proline (IPP), which have been shown to suppress Ang II production and bradykinin catabolism through inhibition of vascular cathepsin-converting enzyme (ACE) activity (31–33).

Correlation studies have shown that there is a strong link between RAS and bone metabolism and that activation of RAS leads to bone loss (34, 35). However, there is no unified view on the target of action and the downstream events of angiotensin II. In the present study, we observed that the expression of AT1R and AT2R was significantly reduced in the bone tissues of osteoporotic mice after intestinal flora dysbiosis, and both losartan and PD123319 could alleviate osteoporosis, but the efficacy of PD123319 was significantly stronger than that of losartan (P = 0.0208), suggesting that the AT2R might be the main target. This seems to synthesize the different results from previous studies, such as those of Shimizu (36) and Zhang (37), who found that Ang II causes osteoporosis through activation of AT1R and by the ERK pathway, and those of Asaba (34), Izu (38) and Monnouchi (39), who confirmed that Ang II is involved in the process of osteoporosis through activation of AT2R involved in the process of osteoporosis. In addition, the significantly stronger efficacy of PD123319 than losartan explains the inability of AT1R inhibitors to alleviate androgenic denervation and osteoporosis due to type 1 diabetes (40).

Interestingly, we observed that the AT2R agonist CGP42112A similarly alleviated osteoporosis caused by gut microbiota dysbiosis. This may be related to the role of AT2R as a “protective arm” that balances pathological processes in the RAS, exerting its protective and reparative effects (41). Furthermore, Lima et al. (42) showed that knockdown of AT2R in healthy mice had a detrimental effect on bone mass, whereas knockdown of AT2R in a periodontitis model had an osteoprotective effect on alveolar bone, suggesting that the role of AT2R may be related to different pathophysiological states (43). However, how the interaction between AT1R and AT2R arises is not clear, and further studies are needed.

In conclusion, our study suggests that antibiotic treatment can lead to gut microbiota dysbiosis and activation of local RAS in bone tissue leading to osteoporosis, a process that promotes RANKL release and inflammatory response through upregulation of ERK1/2 expression, upregulation of osteoclast activity leading to bone loss, and that AT2R may be an important target. However, there are some limitations to this study. First, only one concentration of antibiotic treatment was used in this study, so the relationship between antibiotic treatment and bone loss and RAS could not be fully determined. Second, our findings contradict some studies, and the effect of antibiotic treatment on the gut microbiota still needs to be further investigated. Third, although 16S rRNA sequencing of fecal specimens is currently the main method for assessing the composition of the gut microbiota, it is possible that the fecal microbiota does not reflect the entire gut microbial environment. Fourth, CGP42112A may bind and activate AT1R at high concentrations, which may interfere with the results of the study. Furthermore, we observed antibiotic effects solely on local RAS in bone tissue, preliminarily exploring pathways through which the RAS system participates in osteoporosis mechanisms following dysbiosis. We did not examine other bone metabolism-influencing factors affected by antibiotic treatment, such as short-chain fatty acids, BMP signaling, or physical activity levels. Therefore, further studies are required to elucidate the underlying mechanisms linking RAS to osteoporosis following gut microbiota dysbiosis and to clarify the potential roles of SFACs, BMP signaling pathways, and other factors.

## Data Availability

The original contributions presented in the study are included in the article/supplementary material. Further inquiries can be directed to the corresponding authors.
